# Development of an improved polykaryon-based influenza virus rescue system

**DOI:** 10.1186/1472-6750-12-69

**Published:** 2012-09-25

**Authors:** Vincent Bourret, Jon Lyall, Mariette F Ducatez, Jean-Luc Guérin, Laurence Tiley

**Affiliations:** 1 Cambridge Infectious Disease Consortium, Department of Veterinary Medicine, University of Cambridge, Madingley Road, Cambridge, CB3 0ES, UK; 2 INRA, UMR 1225, IHAP, Toulouse, F-31076, France; 3 Université de Toulouse, INP, ENVT, Toulouse, F-31076, France; 4 Department of Veterinary Medicine, University of Cambridge, Madingley Road, Cambridge, CB3 0ES, UK

**Keywords:** Influenza, Virus rescue, Cell fusion, Reverse genetics

## Abstract

**Background:**

Virus rescue from transfected cells is an extremely useful technique that allows defined viral clones to be engineered for the purpose of rational vaccine design or fundamental reverse genetics studies. However, it is often hindered by low primary rescue success rates or yields, especially with field-derived viral strains.

**Approach:**

We investigated the possibility of enhancing influenza virus rescue by eliciting cell fusion to increase the chances of having all necessary plasmids expressed within the same polykaryon. To this end we used the Maedi-Visna Virus envelope protein which has potent fusion activity in cells from a wide range of different species.

**Results:**

Co-transfecting cells with the eight plasmids necessary to rescue influenza virus plus a plasmid expressing the Maedi-Visna Virus envelope protein resulted in increased rescue efficiency. In addition, partial complements of the 8-plasmid rescue system could be transfected into two separate populations of cells, which upon fusion led to live virus rescue.

**Conclusion:**

The simple modification described here has the potential to improve the efficiency of the virus rescue process and expand the potential applications for reverse genetic studies.

## Background

Influenza is a contagious disease that represents a serious health threat to humans and other animals worldwide. Influenza A viruses, in particular, can infect a variety of species. A global reservoir for these viruses exists in wild waterfowl and shorebirds, from which novel viruses can emerge to infect mammalian species. Influenza is therefore a potential threat to humans, pigs, horses, sea mammals, ferrets, mink as well as many terrestrial bird species [1]. In the past decades, several dramatic episodes of large-scale mortality have occurred in domestic birds, humans, and other species. To date, the main approach to control influenza epidemics and pandemics in human and other animal populations is through vaccination (reviewed in [2]) and biosecurity, although other approaches may help prevent the transmission of highly pathogenic influenza in some species [3].

Strategies for regenerating RNA viral genomes from plasmid DNA clones (“virus rescue”) have enabled the powerful technique of reverse genetics to be applied to many different RNA viruses including, among others, influenza, rabies, Coronavirus, Rift Valley fever virus or fish RNA viruses [4-8]. This technique allows clonal virus stocks with a defined genotype to be engineered. It therefore has major medical applications as it enables vaccines to be rationally designed by inserting attenuating mutations or chosen antigens into a defined viral background, enhancing our control options for pathogens such as influenza virus [9-15]. In fundamental virus studies, it also enables researchers to test the consequences of defined genetic differences on phenotype, such as growth properties, cell-virus interaction, replication cycle characteristics, pathogenicity, or others - the approach known as “reverse genetics”. Various solutions have been developed for satisfying the requirements for the transcribed RNAs to become infectious when produced inside a permissive cell. Among the most complex and demanding systems is that required for the segmented negative stranded RNA viruses such as influenza virus.

The basic strategy for the most commonly used influenza virus rescue systems comprises a set of plasmids that each drives the expression of one viral genome-sense transcript corresponding to each of the eight viral segments. Accurately tailored 5' and 3' termini are achieved by positioning RNA Polymerase I (Pol I) transcription initiation and termination sites respectively (or by using the Hepatitis Delta virus ribozyme to cleave the correct 3' end). Because the RNA is negative sense, the virus polymerase must be produced before replication can ensue. This can be achieved by co-transfecting a further four plasmids that produce the replication proteins (PB2, PB1, PA, NP) in the so-called “12-plasmid system” [16,17]. A derivative of this system uses tandem opposing RNA Pol I and Cytomegalovirus (CMV) promoters to drive expression of viral genome RNAs and mRNAs respectively encoding all the viral proteins, commonly referred to as the “8-plasmid system” [18,19]. Another development has been the production of vectors carrying multiple genome segment expression cassettes [20].

These strategies can be effective and have proven very useful for a number of purposes. However regenerating field strains and some mutants and reassortants is often less efficient than for well-adapted laboratory strains and it is not uncommon for several attempts to be needed before a particular viral construct is successfully rescued. This suggests the existence of one or more limiting factors in the process and prompted us to test whether some of these could be overcome to increase rescue efficiency. Several factors may impact virus rescue efficiency. Examples include the replicative ability of a field-derived avian virus in mammalian cells or the balance of the virus’ haemagglutinin and neuraminidase activities [21]. We hypothesized that one limitation might be the ability to successfully transfect and simultaneously express eight different plasmids within an individual cell. We therefore reasoned that cell fusion might maximize the likelihood of all eight influenza gene-bearing plasmids being present in the same polykaryon and thereby enhance rescue efficiency.

**Figure 1:**
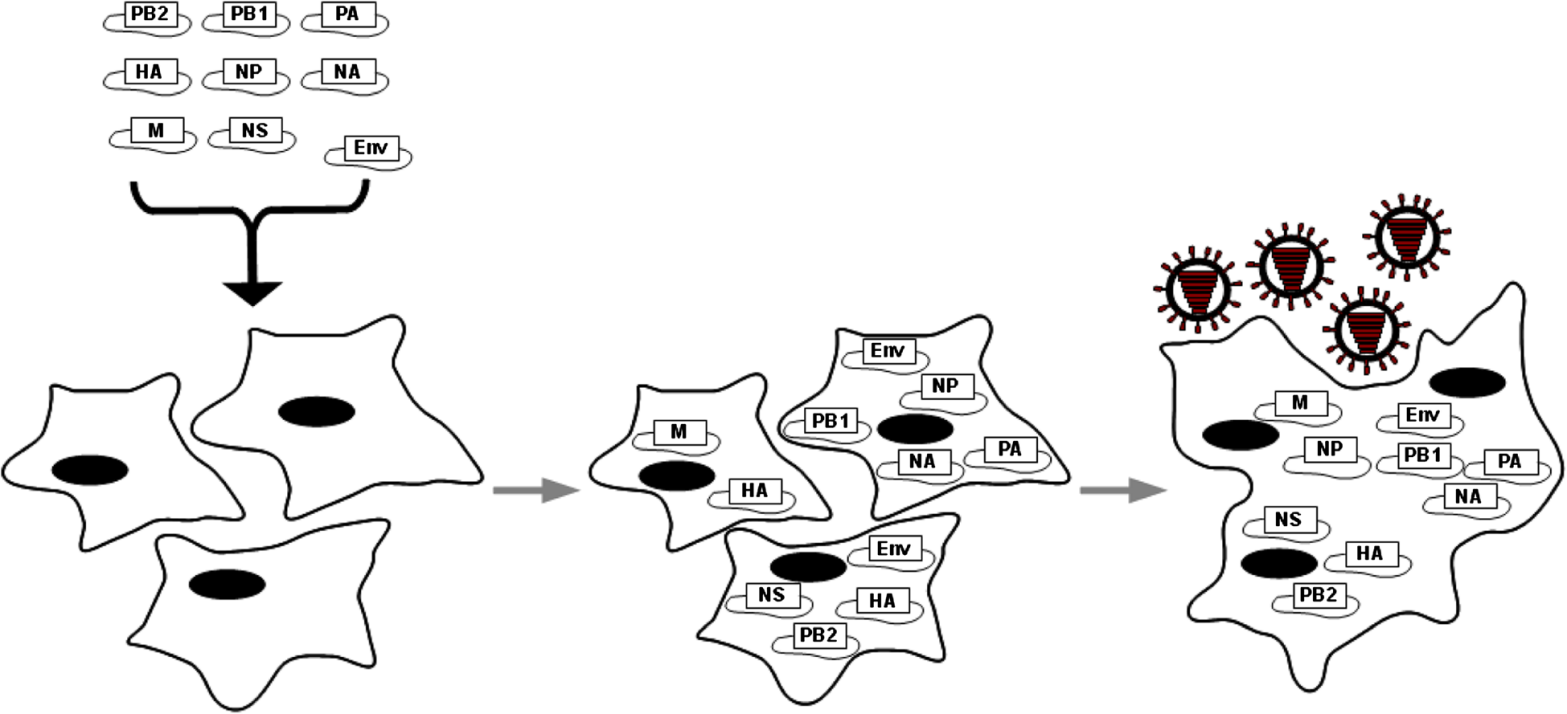
**Principle of the fusion approach applied to the influenza A 8-plasmid rescue system.** The eight virus rescue plasmids are transfected in 293 T cells together with the Env expressing plamsid. Our hypothesis is that individual 293 T cells tend to be transfected with partial complements of the viral rescue plasmids. Upon fusion, all eight necessary viral rescue plasmids can be expressed in one syncytium, allowing rescue of complete virions.

The Maedi-Visna virus (MVV) is a Lentivirus causing chronic pneumonia or a progressive demyelinating disease in sheep. Its envelop glycoprotein (hereafter referred to as Env) is a major target for virus neutralization and can cause substantial host cell fusion. Receptor to this protein has not yet been identified but is known to be expressed on cells from a wide range of species including primates, avians and rodents [22]. We therefore examined co-transfecting an Env expression vector along with the eight plasmid rescue system into HEK 293T/17 cells to determine its effect on virus rescue efficiency (Figure 1).

## Results

### Cell fusion with the Maedi-Visna virus envelope protein (Env) in 293 T

Additional file 1 (Video 1) and Additional file 2 (Video 2) show 293 T cells transfected with 0.4 μg of a Green Fluorescent Protein(GFP)-expressing plasmid plus 0.4 μg of an inert plamid (Video 1), or 0.4 μg of the GFP plasmid plus 0.4 μg of the Env-expressing plasmid (Video 2). Cells were kept in FCS-containing medium for the whole duration of the footage. The video shows a marked fusion process, including visible cell-to-cell GFP transfer, in cells transfected with the Env plasmid, while cells keep dividing over time (Additional file 1: Video 1 and Additional file 2: Video 2).

### Enhanced yields with the Maedi-Visna virus envelope protein (Env)

**Figure 2:**
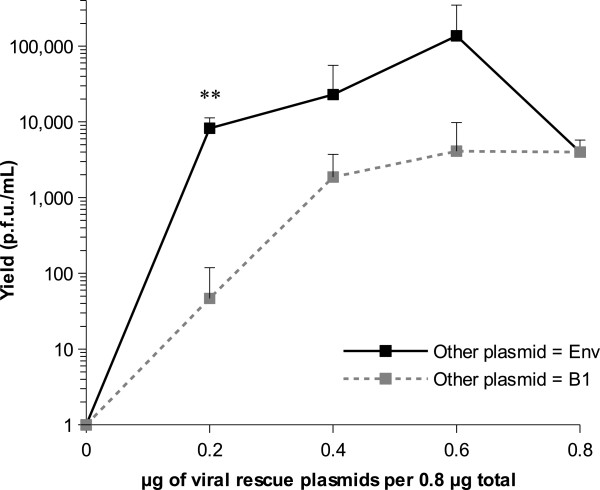
**Yield of rescued PR8 virus obtained using different plasmid proportions.** The total amount of plasmid transfected per well was set to 0.8 μg in a 24-well plate format for the whole experiment. A proportion of this amount was dedicated to the viral rescue plasmids, while the rest of the amount was dedicated either to the Env-expressing plasmid (black symbols) or the B1 inert plasmid (grey symbols), referred to as "other plasmids" in the chart. Each point shows the average yield from three replicate transfections and error bars indicate standard deviation. Negative results (0 p.f.u./mL) are plotted as 1 p.f.u./mL on the logarithmic scale. **, p < 0.01 at the 0.2 μg level.

For virus rescues, we initially worked with a fixed total mass of 0.8 μg of DNA per well in a 24-well plate format, which had previously been determined to be the optimal amount of DNA for best transfection efficiency in our lab. We varied the proportion of viral genes-containing (hereafter “viral rescue” plasmids) and Env-expressing (or an inert control named B1) plasmids comprising the 0.8 μg total in 0.2 μg (25%) increments. Results of this titration for the lab-adapted strain A/Puerto Rico/8/34 (hereafter PR8) are shown in Figure 2. Transfecting 0.8 μg of the viral rescue plasmids alone resulted in an average yield of 4.0x10^3^ ± 1.8x10^3^ (SD) plaque forming units (p.f.u.) per millilitre. Reducing the amount of viral rescue plasmids by replacing them proportionately with the B1 control plasmid exhibited an initial plateau but reduced the yield sharply at the 0.2 μg level. Co-transfecting the Env plasmid tended to increase the yield at all levels and had a particularly marked effect when the viral rescue plasmids were limiting (*e.g.* producing 8.2x10^3^ ± 3.1x10^3^ (SD) p.f.u./mL when as little as 0.2 μg viral rescue plasmids were included; t_4_ = −4.63, p = 0.0098). Increasing the proportion of viral rescue plasmids while decreasing the proportion of Env-expressing plasmid gave higher yields until an apparent optimum was reached for 0.2 μg (25%) of Env-expressing plasmid and 0.6 μg (75%) of viral rescue plasmids. Dispersion of rescue yield values was however generally high and statistical significance was not detected for the differences observed at the 0.4 μg and 0.6 μg levels.

### Application to the rescue of a strain derived from a field sample

**Figure 3:**
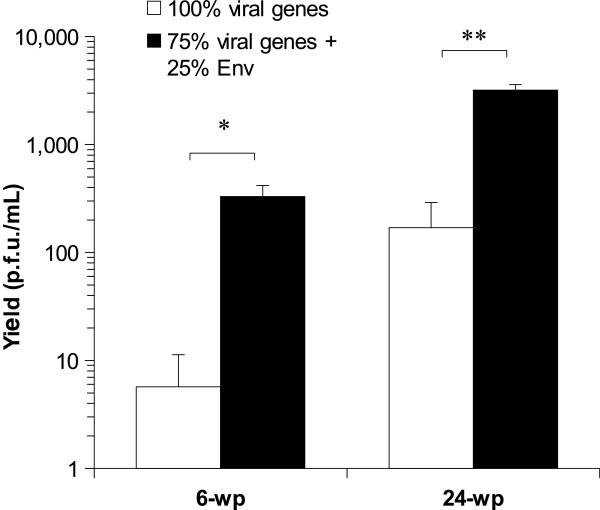
**Effect of using the MVV Env on recovered virus yield of a field-derived LPAI strain.** This figure shows the effect of substituting 25% of the viral genes containing plasmids for the MVV envelope gene-containing plasmid on the rescue efficiency of a low pathogenicity avian influenza strain derived from a field sample. The total amount of transfected DNA is 4 μg per well in 6 well plates and 0.8 μg per well in 24 well plates. The average of three replicates is shown and error bars indicate standard deviation. Asterisks indicate levels of statistical significance as follows: *, p < 0.05; **, p < 0.01. wp, well plate.

We tested whether using the Env-expressing plasmid in the proportions determined above would enhance the practical rescue of a viral strain derived from a low pathogenicity avian influenza (LPAI) field sample (A/mallard/Netherlands/10/99). Such strains are usually more challenging to rescue than laboratory adapted strains such as PR8, giving low yields or failing to rescue in all attempts. In this experiment, Lipofectamine LTX (Invitrogen) was used instead of FuGene 6 (Roche) as transfection reagent as the latter had been discontinued by the manufacturer. Figure 3 shows the comparison of yields when using 100% of viral rescue plasmids versus using 75% of viral rescue plasmids plus 25% of Env-expressing plasmid. This experiment showed an average yield increase of between one and two log in 6-well plates (p = 0.019) and in 24-well plates (p = 0.002) when using fusion. A 0.8 μg total DNA mass was used per well in 24-well plates whereas a 4 μg total DNA mass was used per well in 6-well plates. In 6-well plates, one out of three replicates where fusion was not used was negative (*i.e.* gave no p.f.u.).

### Fusion rescue of incomplete combinations of viral segments

To test whether virus could be rescued from cells containing incomplete complements of viral segments by fusing with cells carrying the missing components, we transfected cells in separate batches with partial complements of PR8 rescue plasmids and then co-cultured them. In an experiment named “4 + 4”, 0.15 μg of each one of segments 1, 2, 3, and 5 as well as 0.2 μg of either B1 or Env-expressing plasmids were transfected into one batch of cells, and identical amounts of segments 4, 6, 7, and 8 as well as 0.2 μg of either B1 or Env-expressing plasmids were transfected into another batch of cells. In another experiment named “7 + 1”, 0.086 μg of each one of segments 1, 3, 4, 5, 6, 7, and 8 as well as 0.2 μg of either B1 or Env-expressing plasmids were transfected into one batch of cells, and 0.086 μg of segment 2 as well as 0.714 μg of either B1 or Env-expressing plasmids were transfected into another batch of cells. On the day after transfection, media were removed, cells were washed once with PBS and then pooled, and on day 3 treated with trypsin. Infectious yields were assessed by plaque formation assay.

Table 1 shows successful rescue of live virus in all attempts where cells transfected separately with complementary sets of influenza rescue plasmids were allowed to fuse together at day 1 post transfection. No live virus was recovered when the empty B1 plasmid was used instead of the Env-expressing plasmid, except for one 100 μL aliquot from the “7 + 1” experiment which yielded one p.f.u.

**Table 1:** Yields (in p.f.u./mL) from cells transfected with partial complements of the eight PR8 rescue plasmids

	**"4+4"**^**1**^		**"7+1"**^**1**^	
	**with B1**^**2**^	**with Env**^**2**^	**with B1**^**2**^	**with Env**^**2**^
Repeat 1	0	5000	0	1400
Repeat 2	0	390	10	50
Repeat 3	0	110	0	150

This table shows the viral yields (p.f.u./mL) from cells transfected with the eight PR8 rescue plasmids split into separate batches at the time of transfection and brought together on the next day.

^1^ In the "4 + 4" experiment, one batch of cells was transfected with plasmids encoding segments 1, 2, 3, 5, while a complementary batch of cells was transfected with plasmids encoding segments 4, 6, 7, 8. In the "7 + 1" experiment, one batch was transfected with plasmids encoding segments 1, 3, 4, 5, 6, 7, 8 and the complementary batch was transfected with segment 2. The cells from complementary batches were dislodged and brought together at day 1 post transfection. These experiments consisted of triplicate transfections for each condition (Repeats 1 to 3).

^2^ In addition to the transfection with influenza plasmids, cells were transfected with either the Env-expressing or the empty B1 plasmid.

### Addition of extra Env-expressing plasmid

**Figure 4:**
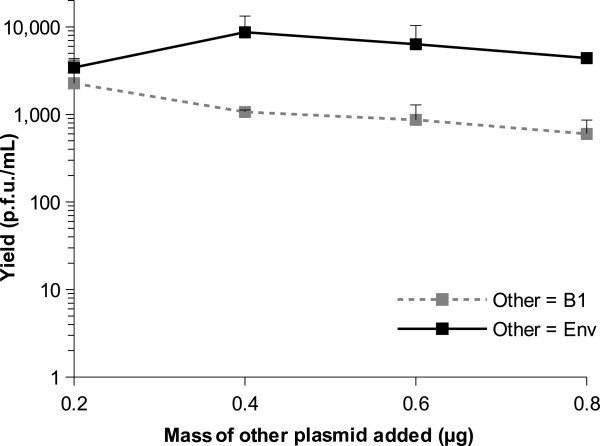
**Effect of adding Env-expressing or inert plasmid beyond 0.8 μg total DNA on primary rescue yields.** This figure shows the effect of adding increasing amounts of Env-expressing or inert B1 plasmid to a fixed amount of 0.6 μg of PR8 rescue plasmids on primary rescue efficiency in a 24-well plate format. Each point shows the average yield from three replicate transfections and error bars indicate standard deviation.

We tested whether adding Env-expressing plasmid beyond a total amount of DNA of 0.8 μg per well in a 24-well plate would be detrimental or beneficial to the rescue. Figure 4 shows the result of a titration where a fixed amount of 0.6 μg of PR8 rescue plasmids was used in addition to which Env-expressing plasmid was added in quantities varying from 0.2 μg to 0.8 μg. The total amount of DNA transfected was therefore 0.8 μg to 1.4 μg per well. Transfection reagent was adjusted to maintain the appropriate ratio to DNA quantity. In order to look solely at primary yield, trypsin was not added during this rescue and viruses were harvested at day 3 post transfection.

This experiment confirmed that in the absence of fusion, adding DNA beyond 0.8 μg per well is detrimental to the rescue, as shown by decreasing yields when adding increasing amounts of the inert plasmid B1, probably due to toxicity or origin competition. However, adding Env-expressing plasmid to PR8 rescue plasmids beyond a total of 0.8 μg of DNA per well gave a modest increase in yields, with a maximum for 0.4 μg of Env-expressing plasmid added to 0.6 μg of PR8 rescue plasmids.

## Discussion

### Increased rescue efficiency with the Env-expressing plasmid

Virus rescue has major applications in the rational design of influenza A vaccines [12,15,23-25], which are to date the main control options in use against this disease. It is also a powerful technique in fundamental research on influenza A, B and C [4,26-28] and many other RNA viruses [29]. It is useful in this context to enhance the technique and improve our ability to rescue viruses bearing a variety of gene combinations. This report shows that cell fusion mediated by the Maedi-Visna virus envelope protein can be an effective tool in overcoming the challenge of having eight different plasmids expressed together in a single cell for the purpose of influenza A virus rescue.

Virus rescue yields remain highly variable as shown by yield variability between replicate transfections observed also when using fusion. However, using fusion increased yields which can help maximize the chances of rescuing some constructs bearing relevant specific gene combinations that make them difficult to rescue. We showed here that it is possible to use up to 0.4 μg Env-expressing DNA together with 0.6 μg of viral rescue DNA in a 24-well plate format to optimize rescue efficiency. We have found that these conditions improved our ability to rescue some artificially reassorted strains bearing specific gene combinations that we had previously not been able to rescue (unpublished results). The difference in transfected DNA concentration between 24- and 6-well plate formats may explain the apparently better virus yield observed in 24-well plates (Figure 3). Cell fusion also allows virus rescue using only a fraction of the amount of cloned viral genes that would normally be required (Figure 2), a potential advantage when large numbers of different constructs have to be made or where some genes that are challenging to clone are available in limiting quantity.

Cell fusion may enhance influenza virus rescue through different mechanisms. We assumed that one limiting factor was the requirement to successfully deliver eight separate plasmids to the same cell and anticipated that this could be enhanced by using cell fusion. This is supported by the “4 + 4” and “7 + 1” experiment results (Table 1) where the complement of plasmids was deliberately incomplete. It is also supported by experiments where the concentration of viral genes-carrying plasmids was reduced to limiting levels, and where inclusion of the Env plasmid restored rescue efficiency (Figure 2). Another hypothesis to account for enhanced yield is that the MVV envelope protein from the host cell might be incorporated into the primary progeny virus envelope and facilitate the first round of infection and hence amplification. However we believe the latter to be unlikely as in order for viral RNPs to uncoat from the matrix protein, they must be exposed to the acid environment of the endosome (reviewed in [30]). Direct membrane fusion mediated via MVV Env is expected to release viral cores directly into the cytoplasm without prior acid exposure and thus non-infectious.

### Other applications of the fusion methodology

In one experiment (Figure 4), we used the trypsin dependence of the PR8 virus in cell culture to eliminate the contribution of amplified virus to the overall yield. Firstly, if it is possible to generate sufficient quantities of primary progeny, one can examine the effect of lethal mutations that may otherwise be unable to undergo amplification (for example mutations in viral proteins or genome RNA that impact on packaging). Viral progeny can be analysed directly for genome complement and ability to initiate a first round of infection. Our method can generate about 10^4^ p.f.u./mL of primary progeny in a 24-well plate format, which could be sufficient for such purposes.

A second application results from the potential offered by fusing cells transfected with different complements of virus segments. It may be possible to establish partial infections in two cell populations, then allow fusion and assay the completion of the virus life cycle. This would be a novel tool studying interactions between different segments during the packaging process and trafficking of viral components.

## Conclusion

The simple modification described herein has the potential to improve the efficiency of the virus rescue process and expand the potential application for reverse genetic studies.

## Methods

### Plasmids

The virus rescue constructs were derived from the RF483 plasmid which is modified from pHW2000 [18]. Both the Env-expressing and the inert B1 control plasmids included a T7 bacterial terminator [31] between the CMV promoter and the insert to prevent transcription in the bacteria. To insert this terminator, the two complementary oligonucleotides T7eAS+: **CAAGAGAAAATGTAATCACACTGGCTCACCTTCGGGTGGGCCTTTCTGCGTTTATAAGGAGACACTTT**CCGGAGTACTGG and T7eAB-: TCGACCAGTACTCCGG**AAAGTGTCTCCTTATAAACGCAGAAAGGCCCACCCGAAGGTGAGCCAGTGTGATTACATTTTCTCTTG** (T7e terminator sequence in bold) were annealed together in 10 mM TrisHCl pH 7.6 and 50 mM NaCl buffer in a boiling temperature waterbath left to cool down overnight. The annealed oligonucleotides formed a double stranded insert bearing one cohesive and one blunt end allowing its cloning into RF483 after this vector was digested with HpaI and SalI (New England Biolabs) as per enzyme manufacturer’s instructions.

### Rescue protocol

On day −1, 5x10^5^ HEK 293T/17 cells (hereafter referred to as 293T; ATCC #CRL-11268) were plated in each well of a 6-well plate (9.5 cm^2^) in Dulbecco's Modified Eagle Medium (DMEM) supplemented with penicillin (100 U/mL), streptomycin (50 μg/mL), amphotericin B (2.5 μg/mL), L-glutamine (10 mM), sodium pyruvate (1 mM) and fœtal calf serum (FCS) (10% v/v). On the next day (day 0), each well was transfected with 4 μg DNA total comprising equal proportions of each of the eight viral plasmids combined with a varying proportion of the Env-expressing or empty control (B1) plasmid as indicated in the figure legends. The B1 empty plasmid controls were included to maintain a constant quantity of DNA and SV40 origins to control for non-specific effects on transfection efficiency and origin replication. For transfections, either 3 μL of FuGene (Roche) (Figures 2 and 4, Table 1) or 3 μL of Lipofectamine LTX and 1 μL of Plus Reagent (Invitrogen) (Figure 3, Additional file 1 video 1 and Additional file 2 video 2) were used per μg of DNA as per manufacturer's instructions. Cells were incubated with the transfection mixture overnight at 37°C and 5% CO_2_ in 1 mL DMEM supplemented with L-glutamine (10 mM), sodium pyruvate (1 mM) and FCS (10% v/v). On the following day (day 1), the medium was removed and cells were carefully washed with PBS before being overlaid with 3 mL DMEM supplemented with penicillin (100 U/mL), streptomycin (50 μg/mL), amphotericin B (2.5 μg/mL), L-glutamine (10 mM), sodium pyruvate (1 mM) and bovine serum albumin (BSA) (0.3% w/v) and left to incubate for three more days.

Smaller scale assays used 24-well plates with 10^5^ cells plated per well and transfected in a 0.5 mL medium volume with 0.8 μg DNA total using 3 μL of transfection reagent per μg of DNA. Cells were incubated overnight at 37°C and 5% CO_2_ in 500 μL medium. On day 1, the medium was removed and cells were carefully washed with PBS before being overlaid with 1 mL serum-free, BSA containing medium as above and left to incubate for three more days.

Except where otherwise stated, trypsin was added to 1 μg/mL on day 3. On day 4, supernatants were harvested, aliquoted and frozen at −70°C. In order to visualize cell fusion more easily, we included control wells containing a GFP expressing plasmid with or without the Env-expressing plasmid.

We first tested this approach on virus A/Puerto Rico/8/34 (PR8), and then on a construct derived from the 8 gene segments of the low pathogenicity avian influenza (LPAI) field sample A/mallard/Netherlands/10/99.

Figure 1 summarises the principle of the fusion approach in the context of the influenza A 8-plasmid rescue system. A video footage of the cell fusion process was made using a BioStation IM-Q device (Nikon) and can be accessed online.

### Titration of viral yields

Viral yields in plaque forming units (p.f.u.) were titrated by plaque formation assay in Madin-Darby Canine Kidney (MDCK) cells using the Avicel method with media supplemented with trypsin [32]. The inoculum was also incubated with tryspin during the virus adsorption period.

### Statistical analyses

Average viral yields between different treatments were compared using 2-tailed Student's t-tests following checks for normality and variance homogeneity using Shapiro-Wilk and Levene tests, respectively. All tests were conducted as implemented in the R statistical program [33].

## Competing interests

The authors declare that they have no competing interests.

## Authors’ contributions

JL, VB and LT conceived the experiments. VB, JL and MFD carried out the experiments. VB carried out the statistical analyses. VB, LT, MFD and J-LG prepared the manuscript. All authors read and approved the final manuscript.

## Supplementary Material

Additional file 1 **Video 1: 293T cells transfected with 0.4 μg of GFP + 0.4 μg of inert B1 plasmid.** Cells were kept in FCS-containing medium for the whole duration of the footage, which spans a 27-hour period from 19 h to 46 h post transfection. More details are provided in the article main text. Speed: x 4200. Click here for file

Additional file 2 **Video 2: 293T cells transfected with 0.4 μg of GFP + 0.4 μg Env plasmid.** Cells were kept in FCS-containing medium for the whole duration of the footage, which spans a 27-hour period from 19 h to 46 h post transfection. The video shows a marked fusion process (compare to Additional video 1), including visible cell-to-cell GFP transfer, while cells keep dividing over time. More details are provided in the article main text. Speed: x 4200. Click here for file
